# Molecular prevalence and associated risk factors of *Anaplasma ovis* in Pakistani sheep

**DOI:** 10.3389/fvets.2023.1096418

**Published:** 2023-03-29

**Authors:** Muhammad Naeem, Itzel Amaro-Estrada, Asia Taqadus, Ayman A. Swelum, Abdulmohsen H. Alqhtani, Muhammad Asif, Muhammad Sajid, Asmat Ullah Khan, Aliza Tariq, Summaya Anjum, Adil Khan, Furhan Iqbal

**Affiliations:** ^1^Institute of Zoology, Bahauddin Zakariya University, Multan, Pakistan; ^2^Centro Nacional de Investigación Disciplinaria en Salud Animal e Inocuidad, Instituto Nacional de Investigaciones Forestales, Agrícolas y Pecuarias (INIFAP), Jiutepec, Morelos, Mexico; ^3^Department of Animal Production, College of Food and Agriculture Sciences, King Saud University, Riyadh, Saudi Arabia; ^4^Institute of Molecular Biology and Biotechnology, Bahauddin Zakariya University, Multan, Pakistan; ^5^Shaheed Benazir Bhuto University Sheringal, District Dir, Khyber Pakhtunkhwa, Pakistan; ^6^Department of Botany and Zoology, Bacha Khan University, Charsadda, Khyber Pakhtunkhwa, Pakistan

**Keywords:** *Anaplasma ovis*, sheep, *msp4* gene, Pakistan, phylogeny

## Abstract

**Background:**

Majority of Pakistani population lives in rural areas and raising animals, especially the small ruminants, is their primary source of income. *Anaplasma ovis* is known to infect small ruminants globally and causing significant economic losses to livestock owners, however prevalence of *Anaplasma ovis* has been least investigated from Pakistan despite having a huge sheep population.

**Methods:**

The present study was conducted from June 2021 till December 2021 to report the PCR based prevalence of *Anaplasma ovis* in the blood samples of sheep (*n* = 239) that were collected from District Dera Ghazi Khan in Pakistan.

**Results:**

Out of 239 samples, 30 (12.5%) amplified a 347 bp fragment specific for the *msp4* gene of *Anaplasma ovis*. Represented partial *msp4* gene sequences were confirmed by Sanger sequencing and deposited to GenBank (OP620757-59). None of the studied epidemiological factors (age, sex, breed, size of herd, dogs with herd, and composition of herd) showed an association (*P* > 0.05) with the *Anaplasma ovis* infection in enrolled sheep. Analysis of the amplified partial *mSP4* sequence of *Anaplasma ovis* revealed that this gene is highly conserved as all three sequences were identical and phylogenetically resembled with the *msp4* sequences amplified from small ruminants in China, Kenya, and Germany, Turkey, Portugal, Tunisia and India. In conclusion, for the first time, we are reporting a moderate prevalence of *Anaplasma ovis* prevalence in Pakistani sheep and this data will help in developing the integrated control policies against this newly reported tick-borne disease that is infecting our sheep breeds.

## Introduction

Diseases, including metabolic, infectious and vector bone, are a major constraint to small ruminants and livestock industry as diseases impose financial risks to the livestock owners due to morbidity and mortality of their animals (1, 2). Ecto parasites, especially ticks, are the major source of pathogen transmission to sheep (3, 4). Tick population as seen a substantial increase globally as well as in Pakistan in recent years due to global weather changes and due to introduction of the animal species in environments/countries where they did not exist before (4). This rise in tick population has led to an increase in the incidence of tick borne diseases (TBDs) and hence the economic losses (5).

*Anaplasma* (*A*.) *ovis*, an intra erythrocytic gram negative rickettsial bacterium, that belongs to the genus *Anaplasma*, family Anaplasmataceae that infect sheep, goats and some wild ruminants causing anaplasmosis (6). *Anaplasma ovis* is frequently reported to be transmitted to sheep by ticks belonging to *Ixodes, Dermacentor, Rhipicephalus*, and *Amblyomma* genera (7). In sheep, infection due to *Anaplasma ovis* is very common and chances of infection increases when the weather is hot and dry or when the sheep are already co infected with some other parasite (8). In extreme cases, infection with *A. ovis* can lead to the death of infected animal (6). The most marked clinical signs of anaplasmosis are anemia and jaundice. Usually a persistent infection is developed in those animals that survive the acute phase of disease (9). Doxycycline is the treatment of choice for the treatment of anaplasmosis and all other tick borne rickettsial diseases (10).

Although Dera Ghazi Khan is known for its large sheep population but ovine anaplasmosis has never been reported from this District. Hence the present study was designed to report the molecular prevalence of *A. ovis* in enrolled sheep breeds and to report the association of this infection with the epidemiological risk factors, if any.

## Materials and methods

### Sample and data collection

Randomly selected herds in Dera Ghazi Khan District were targeted to collect 239 blood samples from apparently healthy sheep during June 2021 till December 2021. Enrolled sheep belonged to four breeds: Mundri (*N* = 171), Kajli (*N* = 44), Latti (*N* = 14), and Baluchi (*N* = 10). Following the informed consent from the owners, around 3–5 ml of blood sample was collected by pricking the Jugular vein of each sheep with a disposable syringe into a tube containing 0.5 M EDTA as an anticoagulant. This blood was later on used for the DNA extraction. In order to find out the epidemiological factors that are associated with *A. ovis* infect in enrolled sheep, a questionnaire was filled on the sampling site. Data regarding sex, age, herd size, composition of herd, and dogs present in herd was collected.

### DNA extraction and PCR amplification

DNA was extracted from the collected blood by using a non-organic method as reported by Grimberg et al. (11). A pair of primers, AOF 5'-TGAAGGGAGCGGGGTCATGGG-3' and AOR 5'-GAGTAATTGCAGCCAGGCACTCT-3' was used to amplify a 347 bp fragment specific for *msp4* gene of *Anaplasma ovis* as previously reported by Torina et al. (12). A master mixture of 25 μl was prepared containing 3 mM MgCl_2_, 10X PCR buffer, 5 μl of template DNA, 0.2 mM deoxy ribonucleotide triphosphates, 2 U of Taq DNA Polymerase (Parstous, Iran) and 0.5 mM of each primer. Reaction conditions comprised of an initial denaturation at 95°C for 5 min, 30 cycles of denaturation for 30 s at 94°C, annealing for 30 s at 62°C and extension for 30 s at 72°C and a final elongation for 5 min at 72°C (12).

### DNA sequencing and phylogenetic analysis

Randomly selected PCR products (*n* = 3) were sent to First Base Sequencing Service (Malaysia) for purification and DNA sequencing. The resultant partial *msp4* sequences from *A. ovis* isolates were deposited at GenBank under the accession numbers OP620757- OP620759. *Anaplasma ovis* sequences (having 96–100% similarity to the ones generated in present investigation) were download from the GenBank database (https://www.ncbi.nlm.nih.gov/) and all sequences were trimmed to 309 bp to be used in phylogeny. Maximum Likelihood method was applied in MEGA version 11 for evolutionary analysis (13). Kimura 2-parameter model with invariant sites was the top ranking substitution model according to lowest Bayesian Information Criterion score (14). Also, a bootstrap analysis with 1,000 replicates was used for the tree construction. *Anaplasma phagocytophilum*'s *msp4* partial gene sequence was used as an outgroup. Sequence alignment was performed by using ClustalW and visualizated with BioEdit (15).

### Statistical analysis

Minitab (version 17, USA) was used for data analysis. *P* ≤ 0.05 was selected as significant level. Comparison of *A. ovis* prevalence between various sheep breeds was made by applying one way analysis of variance (ANOVA). Association between *A. ovis* occurrence and various risk factors was assessed through the Fisher's exact test (for 2 × 2 tables). Tajima's D Fu and Li's D values were estimated with DnaSP v5 (16).

## Results

### Molecular investigation and risk factors' analysis

Analysis of the results revealed that PCR amplified a 347 base pair fragment specific for *msp4* gene of *A. ovis* in 30 out of 239 (12.5%) collected sheep blood samples during present molecular survey. When prevalence of *A. ovis* was compared between enrolled sheep breed, one way ANOVA results revealed that the bacterium prevalence was not restricted to a particular sheep breed (*P* = 0.3; Table 1). Fisher Exact test results revealed that all the epidemiological parameters investigated during this survey were not associated (*P* > 0.05) with the *A. ovis* infection in sheep (Table 2).

**Table 1:** Comparison of *Anaplasma ovis* prevalence in blood samples of various sheep breeds enrolled from District Dera Ghazi Khan during present study.

**Sheep Breeds**	***N***	***Anaplasma ovis +*ve sheep**	***Anaplasma ovis* −ve sheep**	***P*-value**
Mundri	171	18 (10.5%)	153 (89.5%)	0.3
Latti	14	2 (14.3%)	12 (85.7%)	
Kajli	44	8 (18.2%)	36 (81.1%)	
Baluchi	10	2 (20%)	8 (80%)	
Total	239	30 (12.6%)	209 (87.5%)	

N represents the total number of sheep samples collected from each breed. % prevalence of Anaplasma ovis is given in parenthesis. P-value represents the results of one way ANOVA test calculated for studied parameter.

P < 0.05 = Non-significant.

**Table 2:** Association of *Anaplasma ovis* prevalence with the studied epidemiological parameters describing sheep characters enrolled during the present study from District Dera Ghazi Khan.

**Parameters**		***Anaplasma ovis* +ve sheep**	***Anaplasma ovis* −ve sheep**	**P-value**
Sex	Male	1/9 (11.1%)	8/9 (88.9%)	1
	Female	29/230 (12.6%)	201/230 (87.4%)	
Age	< 2 years	14/123 (11.4%)	109/123 (88.6%)	0.7
	>2 years	16/116 (13.8%)	100/116 (86.2%)	
Composition of herd	Sheep only	18/160 (11.3%)	142/160 (88.8%)	0.4
	Sheep and goat	12/79 (15.2%)	67/79 (84.8%)	
Dogs with herd	Present	7/55 (12.7%)	48/55 (87.3%)	1
	Absent	23/184 (12.5%)	161/184 (87.5%)	
Size of herd	< 20	7/47 (14.9%)	40/47 (85.1%)	0.6
	>20	23/192 (12%)	169/192 (88%)	

N represents the total number of collected samples. % prevalence of Anaplasma ovis is given in parenthesis. P-value represents the results of Fischer Exact test calculated for each studied parameter.

P > 0.05 = Non-significant.

### Phylogenetic study and genetic diversity

**Figure 1:**
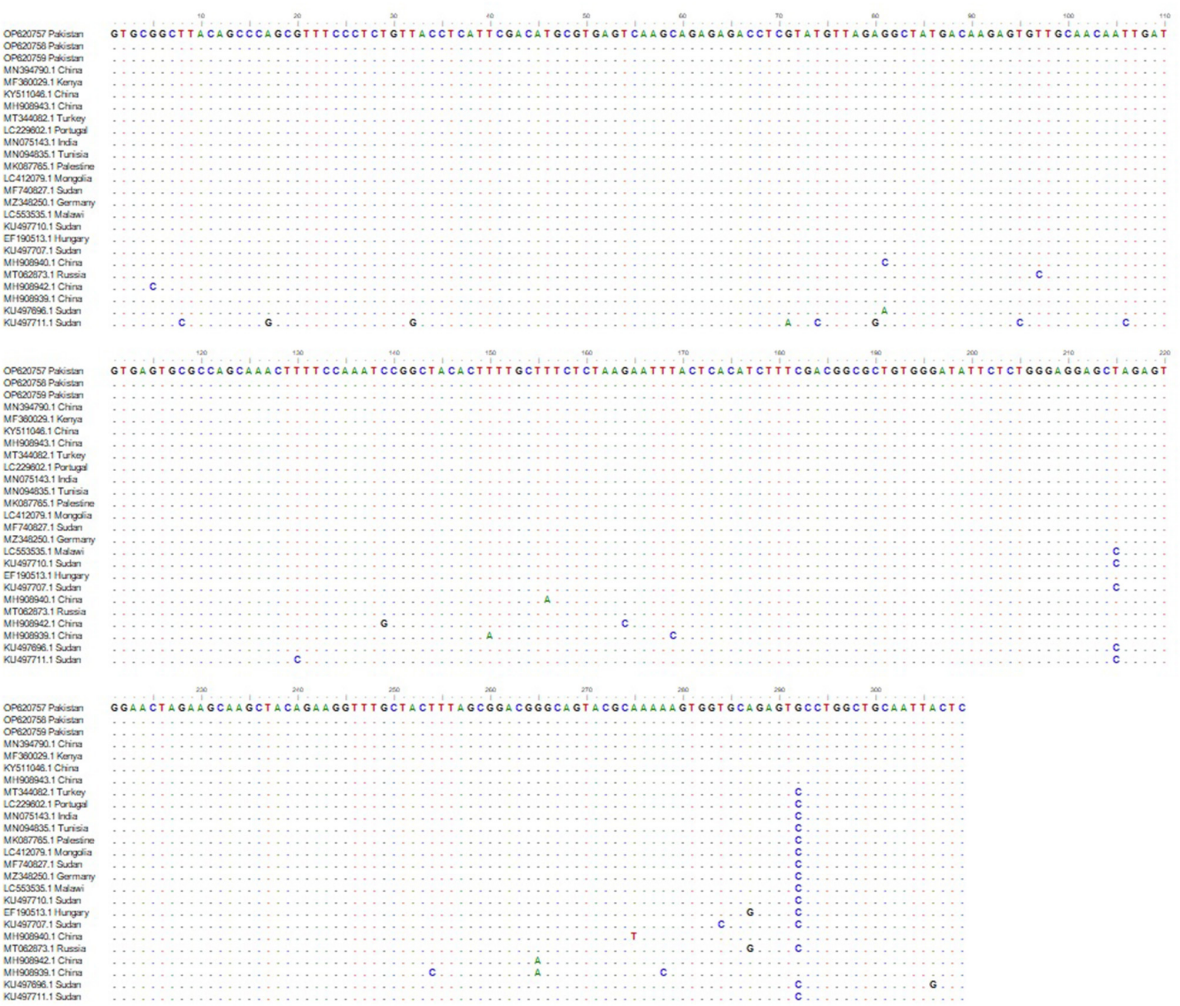
Phylogenetic analysis of *Anaplasma ovis* based on *msp4* partial gene sequence. Sequences in the box were determined in this study. GeneBank accession numbers and country are shown for all sequences. Values at the nodes represent the number of occurrences of clades in 1,000 bootstrap replications of the taxa. The *msp4 partial gene* sequence of *Anaplasma marginale* from Mexico, *Anaplasma centrale* from Israel were and *Anaplasma phagocytophilum* from Italy were used as the outgroup. Maximum Likelihood method and Kimura 2-parameter model were used to infer the evolutionary history. The tree with the highest log likelihood (−1,168.91) is shown. Evolutionary analyses were conducted in MEGA11.

During phylogenetic analysis, we have compared the partial *msp4* (347 bp) gene sequences generated during this study (OP620757–OP620759) with those previously deposited GenBank from various parts of the World having sequence homology of 96% or more (Figure 1). Analysis revealed that all three *A. ovis* sequences generated during present study clustered in a single cluster as shown in Figure 1 along with those amplified from small ruminants in Kenya, China, Germany, Turkey, Portugal, Tunisia, and India (Figure 1).

**Figure 2:**
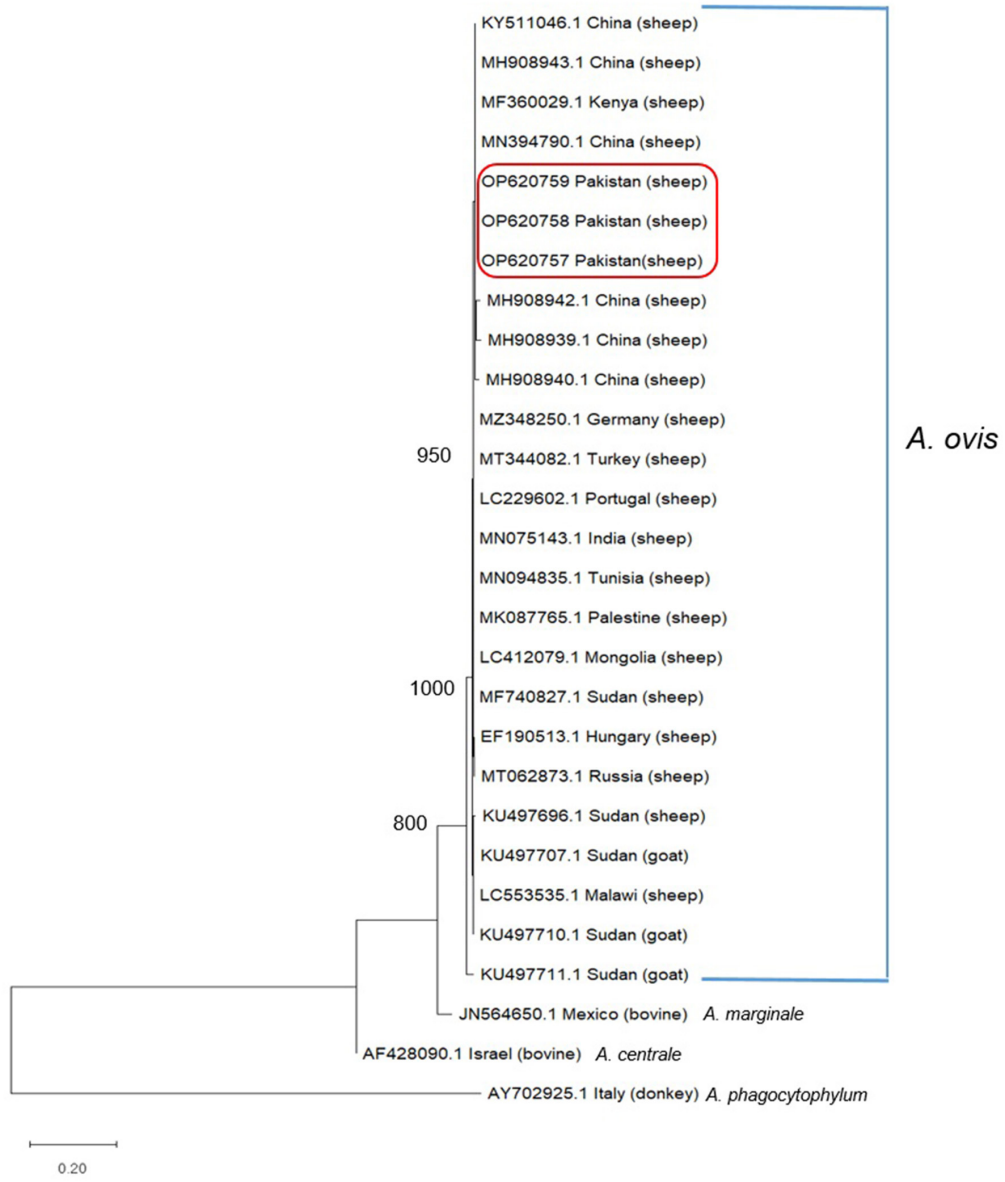
*msp4* sequence alignment from *Anaplasma ovis* isolates amplified from Pakistani sheep and the sequences deposited in GenBank from various parts of world. Dashes indicate the conserved nucleotide positions. The positions with substitutions in DNA sequence of various *Anaplasma* spp. are represented by different colored nucleotides.

Alignment of *msp4* partial sequences of *msp4* gene from Pakistani sheep revealed a single genotype (Figure 2). All the three sequences generated during present study showed 100% genetic similarity with one another indicating that this *msp4* sequence is highly conserved (Figure 2). While these sequences had 96–100% identity with the *msp4* sequences submitted in GenBank from various countries (Figure 2). The phylogeny did not indicate a population structure that was based on geography. Thereof, the set of sequences generated in this study was taken as a single population for neutrality test. Calculated values for Fu and Li's *D*-test was −3.49615 with a statistical significance of *P* < 0.02 and the value for Tajima's *D* was −2.16609 with a statistical significance of *P* < 0.01.

## Discussion

Ovine anaplasmosis is among the most common TBDs reported in sheep from worldwide especially in tropical and sub-tropical regions (17). Since its identification in 1912, *Anaplasma ovis* has been reported from Asia, Europe, Africa, and the United States (8). Usually infection caused by *Anaplasma ovis* is not severe but cases with severe pathology in small ruminants have been documented from Northern United States and from Africa (18). In Haibei County of western China, during 2008, infection due to *A. ovis* resulted in 17% mortality and 40–50% sheep morbidity (19). Due to increased tick infestation, the incidence of ovine and caprine anaplasmosis is rising worldwide (20). Data regarding the prevalence of *A. ovis* in sheep and goats of Pakistan is very limited and only one previous report is available in literature to date.

In present study, we have reported that 12.5% of collected blood samples from sheep were infected with *Anaplasma ovis*. The only study from Pakistan is reported by Niaz et al. (21) in which sheep were enrolled from northern areas of Pakistan and they have used molecular tool (PCR) for the detection of *Anaplasma spp*. and they had found that 21.7% of enrolled sheep were infected with *A. spp*. and DNA sequencing of the amplified PCR products confirmed the presence of *A. ovis* in enrolled sheep. The prevalence of *A. ovis* has been reported from various parts of the world. The prevalence of *A. ovis* was reported to be 70.1% in sheep of Tunisa (22), 69% in sheep of Mogolia (23), 54.5% in sheep of Qinghai, China (24), 34.2% in sheep of central and Western Kenya (25), 29.7% in sheep of Turkey (26), 20.8% in sheep of Iran (27), 10% in sheep of West Iran (28), 5.7% in sheep of South Western China (29), and 2.6% in sheep of North East China (30). The variation in the prevalence of *Anaplasma ovis* between different studies is due to difference in the geographical and climatic conditions of sampling sites, age, gender, immunity of the host animal, tick density in a specific area, and also depends upon type of farm management technique that was followed during a specific study (31).

Phylogenetic and sequence analysis of *msp4* partial sequence amplified during this study revealed that this gene sequence highly conserved (Figures 1, 2). Phylogenetic analysis revealed that our amplified DNA sequences are placed in stable monophyletic with 100% homology with *msp4* partial sequences from China, Kenya, Germany, Turkey, Portugal, Tunisia, and India (Figure 1). However, our isolates are relatively distant from the strains isolated from small ruminants in Sudan, Hungry and Russia. The genetic variations between *msp4* sequences of *A. ovis* that we have generated and those deposited in GenBank are probably due to the difference of geographic conditions of the areas from the bacterium samples were detected as not only the tick diversity and density varies with the geographic and climatic conditions but also the pathogenicity of *A. ovis* strains is also affected (32). Negative values of Tajima's *D* and Fu's *F* were obtained during present study that indicates an excess of rare variation, deviations from neutrality and a recent expansion of the population. This is consistent due to the origin of the sequences used. However, there number of DNA sequences that were used in the analysis was limited and use of large number of DNA sequences are recommended in future studies for a thorough evolutionary analysis. Further, we are not excluding that other *msp4* variants may be circulating in the small ruminants of Pakistan.

In present study, all the epidemiological factors (age, sex, breed, size of herd, dogs with herd, and composition of herd studied) were not found associated with the prevalence of *A. ovis*. Contrary to our result, it is reported that eve are more susceptible to *A. ovis* infection as compared to ram. This is because of the fact that eve faces more hormonal fluctuations due to their reproductive cycles that makes them more susceptible to infections (22, 28). It is also reported that adult sheep were more affected than lambs. This is probably due to the fact that adults are more exposed to the environment (for grazing and for marketing) and hence they have higher chances to encounter vectors rather than lambs that are mostly kept at farms (22, 27).

## Conclusions

In conclusion, we are reporting a moderate prevalence of *A. ovis* in sheep blood samples that were collected from Dera Ghazi Khan in Punjab (Pakistan). None of the enrolled sheep breed was specifically susceptible to *A. ovis*. Data generated in this study will pave the way for the prophylactic detection and control of ovine anaplasmosis in Pakistan. We recommend that similar and large scale studies must be conducted in all those areas of Pakistan that are unexplored for the incidence and prevalence of *A. ovis*. This will significantly help in control of tis bacterium and will improve the output of livestock sector in Pakistan.

## Data availability statement

The data presented in the study are deposited in the GenBank, accession numbers OP620757-59.

## Ethics statement

The animal study was reviewed and approved by Ethical Committee of Institute of Pure and Applied Biology, Bahauddin Zakariya University Multan (Pakistan). Written informed consent was obtained from the owners for the participation of their animals in this study.

## Author contributions

FI and AdK designed and supervised the study. MN, MA, and ATar collected blood samples from sheep and noted epidemiological data. MN, MS, AsK, ATaq, and SA extracted DNA from blood samples and carried out PCR assays. IA-E, AS, and AA performed or assisted with the statistical analysis, sequence alignment, and phylogenetic study. MN, AS, AA, and FI wrote the text and edited and finalized the article. All authors approved the final version of the article.
